# Catechin potentiates the antifungal effect of miconazole in *Candida glabrata*

**DOI:** 10.1007/s12223-023-01061-z

**Published:** 2023-05-05

**Authors:** Nora Tóth Hervay, Daniel Elias, Marcela Habova, Juraj Jacko, Marcela Morvova, Yvetta Gbelska

**Affiliations:** 1 Faculty of Natural Sciences, Department of Microbiology and Virology, Comenius University in Bratislava, Ilkovicova 6, Bratislava, 842 15 Slovak Republic; 2 Faculty of Mathematics, Physics and Informatics, Department of Nuclear Physics and Biophysics, Comenius University in Bratislava, Mlynska Dolina, Bratislava, 842 48 Slovak Republic

**Keywords:** Candida glabrata, Antifungal azole, Catechin-hydrate, ROS, Anisotropy

## Abstract

The rising number of invasive fungal infections caused by drug-resistant *Candida* strains is one of the greatest challenges for the development of novel antifungal strategies. The scarcity of available antifungals has drawn attention to the potential of natural products as antifungals and in combinational therapies. One of these is catechins—polyphenolic compounds—flavanols, found in a variety of plants. In this work, we evaluated the changes in the susceptibility of *Candida glabrata* strain characterized at the laboratory level and clinical isolates using the combination of catechin and antifungal azoles. Catechin alone had no antifungal activity within the concentration range tested. Its use in combination with miconazole resulted in complete inhibition of growth in the sensitive *C. glabrata* isolate and a significant growth reduction in the azole resistant *C. glabrata* clinical isolate. Simultaneous use of catechin and miconazole leads to increased intracellular ROS generation. The enhanced susceptibility of *C. glabrata* clinical isolates to miconazole by catechin was accompanied with the intracellular accumulation of ROS and changes in the plasma membrane permeability, as measured using fluorescence anisotropy, affecting the function of plasma membrane proteins.

## Introduction

Many fungal species are part of the normal microbiota found in different anatomical sites of the human body and play an important role in human health (Arastehfar et al. 2020; Rolling et al. 2020). However, when the immune system is impaired, commensal fungal species can turn into invasive pathogens and develop invasive fungal infections. Fungal species belonging to *Candida spp*. are the most clinically relevant pathogens causing invasive fungal infections. Although most candidemia cases are caused by *Candida albicans*, there has been a steady shift towards non-*albicans* species over the past years. Invasive candidiasis due to *C. glabrata* causes substantial morbidity and mortality, perhaps due to the inherent low susceptibility of *C. glabrata* to the most commonly used antifungal azoles (Timmermans et al. 2018). The acquisition of resistance frequently observed with *C. glabrata* has been ascribed to its haploid genome. Only three groups of licensed antifungal drugs are applied for the treatment of life-threatening blood-stream *Candida* infections. These are triazoles (fluconazole, voriconazole, posaconazole), the echinocandins (caspofungin, micafungin, anidulafungin), and polyenes (different formulations of amphotericin B (Antinori et al. 2016)). Recently, echinocandins are considered as the most effective antifungals, but their application is limited by the high cost of echinocandin therapy (Pea and Lewis 2018). Despite the successful introduction and application of the above-mentioned antifungal drug groups in the clinical therapy, *Candida* infections with fatal outcome are becoming more frequent as a consequence of emerging resistance mechanisms (Cleveland et al. 2015).

The increased incidence of invasive mycoses and the problem of antimicrobial resistance together with the limited efficacy of current antifungal agents have motivated the search for new drugs. Natural resources provide many potential bioactive molecules serving as promising alternatives to the conventionally applied antifungal drugs. One group of plant-derived substances—the flavonoids—is capable of promoting many valuable effects on humans. The identification of flavonoids with possible antifungal effects at low concentrations or in synergic combinations with existing antifungals could help to overcome the resistance problem. Catechins, the polyphenolic compounds known as flavanols, are found in a variety of plants. The main dietary sources of these flavanols are a variety of fruits, vegetables, and plant-based beverages, e.g., green tea. Catechins have potent antioxidant properties, although in some cases they may act as pro-oxidants. Catechins can interact with membranes via adsorption or penetration into the lipid bilayers (Fraga et al. 2010). Phenolic structures often have the potential to strongly interact with proteins due to the interaction of their hydrophobic benzene rings with protein proline residues and the hydrogen-bonding potential of the phenolic hydroxyl groups (Fraga et al. 2010). In vitro studies demonstrated the antimicrobial effects of catechins on both gram-positive and gram-negative bacteria, including multidrug-resistant strains (Wu and Brown 2021). Although multidrug resistance to azoles, echinocandins, and polyenes is still uncommon within the *Candida* genus, its emergence in several *Candida* species has been reported and points towards an increasing trend among *C. glabrata* and *C. auris* isolates (Arendrup and Patterson 2017). The aim of the current study was to evaluate the effect of catechin in combination with antifungal azoles in *C. glabrata* laboratory strain as well as in *C. glabrata* clinical isolates*.*

## Material and methods

### Yeast strains, primers, and media

The *C. glabrata* strains used in this study were the following: laboratory strain *Cglig4Δ lig4*::*HIS3 trp1* (Cen et al. 2015), kindly provided by Patrick van Dijck (KU Leuven, Belgium). The *Cglig4Δ* strain in which the *LIG4* gene has been deleted was generated to improve the homologous recombination efficiency in *C. glabrata*. The phenotypic analysis showed that the *lig4* mutant strain behaves exactly as the wild type for all conditions tested (Cen et al. 2015). *C. glabrata* clinical isolates SM1 and azole-resistant clinical isolate SM3 (Whaley et al. 2014) kindly provided by SG. Whaley (University of Tennessee Health Science Center, Memphis, Tennessee, USA). Based on CLSI breakpoints, susceptibility to fluconazole was defined by an MIC of ≤ 8 μg/mL and resistance was defined by an MIC ≥ 64 μg/mL (Magill et al. 2006). Cells were grown in liquid YPD (1% yeast extract, 2% peptone, 2% glucose). For solid media, 2 g/100 mL of agar was added to the liquid medium mentioned.

### Drug susceptibility assays

The susceptibility of *C. glabrata* strains to various cytotoxic compounds was determined by spotting assays. Yeast cultures grown overnight in YPD medium were diluted to a cell concentration of 1.0 × 10^7^/mL, and serial tenfold dilutions were prepared. A total of 5 μL aliquots of cell suspensions were spotted onto solid agar plates, containing the indicated concentrations of drugs. Colony growth was scored after 2 days of incubation at 30 °C. The selected drug concentrations added to the growth medium were as follows: (±)-catechin hydrate (Sigma-Aldrich) 2 mg/mL; miconazole 0.05 µg/mL, 0.5 µg/mL; fluconazole 10 µg/mL. Based on our preliminary studies of the antifungal activity of catechin-hydrate, epicatechin, and epigallocatechin gallate on *C. glabrata* cells that showed a similar antifungal effect of these in combination with antifungal azoles (fluconazole, miconazole), in this work, we used only the catechin-hydrate, named as catechin in the following text, as a representant of all three catechins.

### Fluorescence anisotropy measurements

The cells grown in YPD medium at 30 °C to the mid-exponential phase were washed twice in Tris–Cl buffer (10 mmol/L, pH 7.0). The cells (A_600_ of 0.1) were labeled with DPH or TMA-DPH, in the final concentration of 1.5 × 10^−7^ mol/L. Plasma membrane fluidity was determined using the Luminescence Spectrometer Perkin Elmer LS 55 with L-format measurement. The excitation wavelength was 360 nm, and the emission wavelength was 430 nm. Anisotropy (rs) was calculated as described in Bencova et al. (2020). For statistical analyses, the one-way analysis of variance (ANOVA) and post hoc Dunnett multiple comparisons with control were used (unpaired t-test).

### Quantitative real-time PCR

Total RNA was extracted from exponentially grown cells as described previously (Bencova et al. 2020) and used to quantify the expression of the *CgCDR1* gene encoding the main *C. glabrata* efflux pump. First-strand cDNA was synthesized from 1 μg of total RNA using oligo dT(18) and Revert AID™ H Minus M-MuLV Reverse Transcriptase (Thermo Fisher Scientific, Frankfurt am Main, Germany). Quantitative real-time PCR was performed in triplicate as described previously (Bencova et al. 2020). Primers used to perform RT-PCR experiments are listed in Table 1.

**Table 1:** List of oligonucleotides used in this study

	Forward	Reverse
*ACT1*	TTCAACGTTCCAGCCTTCT	GTAACACCGTCACCAGAGT
*CDR1*	TGGACCCTACTTCCGATGAG	GCGACCAAATCCTTCCAGTA

### Rhodamine 6G efflux

Active efflux of rhodamine 6G (Sigma-Aldrich, Taufkirchen, Germany) was determined as described in Gbelska et al. (2017). Yeast cells were grown in 10 mL of YPD medium at 30 °C for 20 h. 5 × 10^8^ cells from an overnight culture were incubated in 100 mL of YPD medium and grown for 2 h at 30 °C. About 10^9^ cells were pelleted and washed three times with 50 mmol/L HEPES/NaOH, pH 7.0. Cells were resuspended in 50 mmol/L HEPES/NaOH containing 2 mmol/L 2-deoxyglucose and 10 μmol/L rhodamine 6G and shaken for 2 h at 30 °C to exhaust the energy and allow rhodamine 6G accumulation. Cells were then washed three times and resuspended in 50 mmol/L HEPES/NaOH, pH 7.0, to a cell concentration of 10^8^ per mL. At a specific time interval after the addition of glucose (final concentration, 2 mmol/L) to initiate rhodamine 6G efflux, the cells were centrifuged, and 100 μL supernatants were added to Nunc 96-well fluoro-/luminunc plates (Nagle Nunc International, Rochester, NY). Rhodamine 6G fluorescence of the samples was determined using a Varioscan Flash spectrofluorimeter (Thermo Fisher Scientific, USA) at the excitation wavelength of 515 nm and the emission wavelenght of 555 nm.

### Detection of intracellular ROS levels

The production of ROS was measured using dihydrofluorescein diacetate (H_2_DCFDA) which produces fluorescence after being attacked by ROS (Okai et al. 2000). The cells were grown to the late exponential phase in YPD. 1 × 10^9^ cells in 10 ml of YPD were pretreated with catechin (2 mg/mL), miconazole (0.5 μg/mL), or with both chemicals for 2 h at 30 °C. Cells were washed in phosphate-buffered saline (PBS). A suspension of 1 × 10^5^ cells was prepared in PBS and incubated with 25 μM H_2_DCFDA (Sigma-Aldrich, dissolved in DMSO) in a 96-well plate. The DCF fluorescence signal was measured using the GloMax Discover Microplate Reader (Promega Corp.) at 0, 30, 60, and 90 min at excitation and emission wavelengths of 475 and 500–550 nm, respectively.

## Results

**Fig. 1:**
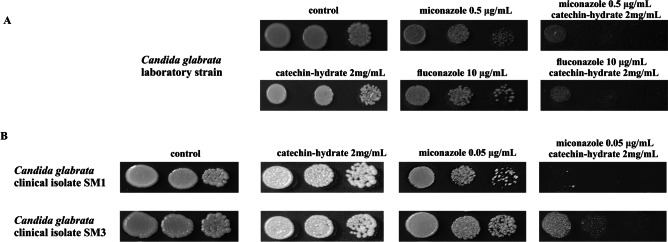
Susceptibility of *C. glabrata* wild-type strain (**A**) and the *C. glabrata* clinical isolates (**B**) to miconazole and fluconazole alone and in combination with catechin-hydrate. Cells were spotted as tenfold dilution series on YPD plates and incubated at 30 °C for 2 days

*Candida glabrata* represents a major threat to global health as resistance to multiple classes of antifungal drugs is common. Inspired by in vitro studies demonstrating the antimicrobial effects of catechins on both gram-positive and gram-negative bacteria, we evaluated the possible synergism of the combination of catechin and antifungal azoles against *C. glabrata* laboratory strain as well as azole-sensitive and azole-resistant clinical isolates. Figure 1 shows that the growth of all *C. glabrata* strains in the presence of catechin was similar as that in the control YPD medium. However, the addition of catechin together with fluconazole or miconazole resulted in the enhancement of the antifungal activity of both antifungal azoles in the laboratory *C. glabrata* strain. The effect of the combined antifungal activity of miconazole and catechin against azole susceptible *C. glabrata* clinical isolate is shown in Fig. 1B. The growth of clinical isolate was completely inhibited using miconazole (0.05 µg/mL) together with catechin (2 mg/mL) compared with miconazole alone. Figure 1B shows that the combined use of miconazole and catechin yielded significant growth inhibition also in the azole-resistant *C. glabrata* clinical isolate.

### Increased intracellular ROS generation induced by cotreatment with catechin and miconazole in *C. glabrata*

**Fig. 2:**
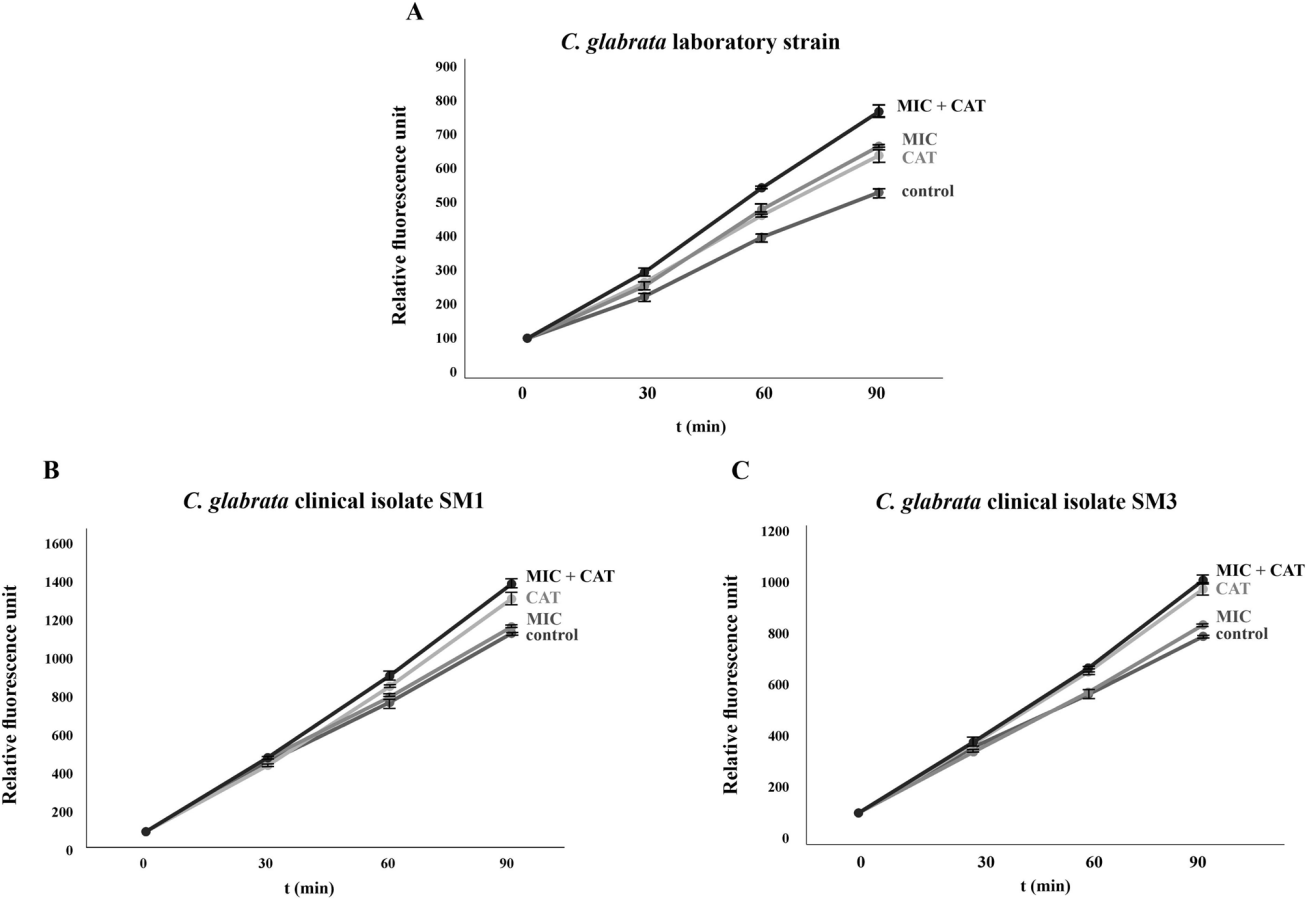
Production of ROS by *C. glabrata* wild-type strain (**A**) and *C. glabrata* clinical isolates: sensitive to antifungal azoles (**B**) and resistant to antifungal azoles (**C**) in the presence of miconazole (0.05 µg/mL for the clinical isolates and 0.5 µg/mL for wild-type strain), catechin-hydrate (2 mg/mL), and miconazole with catechin-hydrate (0.05 or 0.5 µg/mL + 2 mg/mL)

Previous reports have shown that catechins appear to be able both to generate and to scavenge free radicals (Bernatoniene and Kopustinskiene 2018). Antifungal azoles also induce the accumulation of reactive oxygen species (ROS) in fungi. The presence of intracellular ROS in *C. glabrata* strains was assessed using the fluorescent probe H_2_DCFDA-2′,7′-dichlorodihydrofluorescein diacetate. We evaluated the production of ROS in *C. glabrata* strains after the challenge with miconazole or catechin alone and with the combination of both miconazole and catechin. As Fig. 2A shows, both miconazole- and catechin-challenged cells produced an increased amount of ROS in the *C. glabrata lig4Δ* strain compared to the control. The simultaneous presence of miconazole and catechin induced an even higher proportion of ROS in cells of the *C. glabrata lig4Δ* strain compared with the compounds alone. Although in both *C. glabrata* clinical isolates, the combined use of miconazole and catechin induced the highest amount of ROS, the amount of ROS was comparable with the amount accumulated by the presence of catechin alone (Fig. 2B, C). Figure 2B clearly shows that the amount of induced ROS is higher in the susceptible *C. glabrata* clinical isolate compared to the resistant one (Fig. 2C).

### Plasma membrane fluidity

**Fig. 3:**
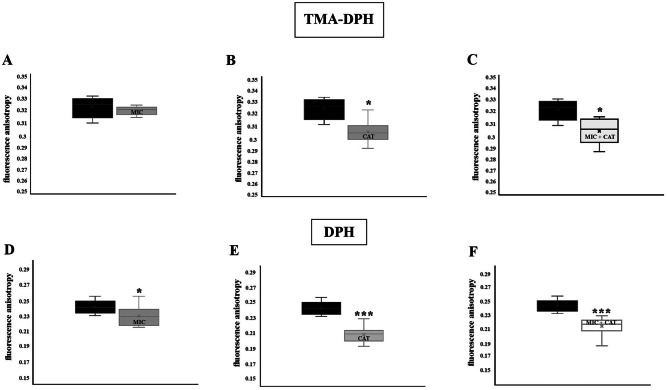
TMA-DPH (**A**, **B**, **C**) and DPH (**D**, **E**, **F**) fluorescence anisotropy of *C. glabrata* wild-type strain in the presence of miconazole (0.5 µg/mL) (**A**, **D**), catechin-hydrate (2 mg/mL) (**B**, **E**), and in the combination of miconazole with catechin-hydrate (**C**, **F**). The mean—the band inside the box, the bottom and top of the box represent standard deviation, and the ends of the whiskers represent the minimum and maximum obtained value. The values represent the mean of 10 independent biological replicas

Beside the ROS induction, catechins can intercalate into the lipid bilayer, leading to lateral expansion and altering cell membrane permeability (Sun et al. 2009). To investigate whether the increased susceptibility of cells treated with miconazole and catechin is attributable to changes in plasma membrane fluidity, we measured the fluorescence anisotropy of whole cells using TMA-DPH and DPH as probes. The polar region of the TMA-DPH probe anchors at the lipid-water interface, whereas the hydrocarbon moiety enters the lipid part of the membrane (Prendergast et al. 1981; Kuhry et al. 1983). The TMA-DPH probe thus provides information on the more superficial region of the plasma membrane. The DPH probe incorporates into the hydrophobic regions of the lipid bilayer, and measurement of its anisotropy correlates with membrane integrity or the ordering of lipid molecules (Lakowicz 2006; Sharma 2006). Fluorescence anisotropy in the *C. glabrata lig4Δ* strain grown in the presence of catechin was significantly lower compared to that measured in the presence of miconazole (Fig. 3B, E, and Fig. 3A, D). The lower the anisotropy, the more fluid the membrane. The plasma membrane of cells incubated in the presence of catechin is therefore more fluid.

### Expression and function of CgCdr1p in the presence of catechin

**Fig. 4:**
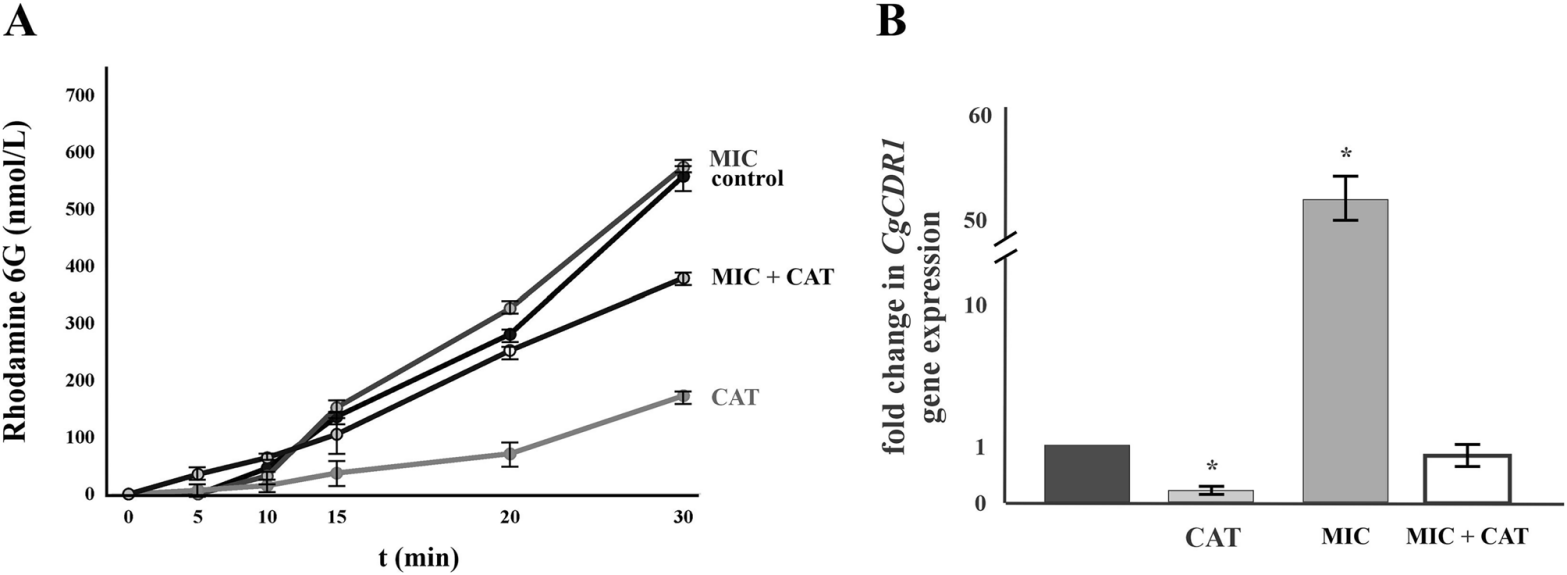
Energy-dependent rhodamine 6G efflux (**A**) and relative levels of *CgCDR1* gene expression (**B**) of *C. glabrata* wild-type strain in the presence of miconazole (0.5 µg/mL) and catechin hydrate (2 mg/mL) alone or in the combination of miconazole with catechin-hydrate (0.5 µg/mL and 2 mg/mL). The gene transcript level in the wild-type strain was set as 1. The results are the mean ± SD for three independent experiments. Significance: * *p* < 0.05. Values were calculated via the student’s t-test and indicate significant differences between the control cells (no induction) and the cells incubated with catechine-hydrate, miconazole, or their combination

Changes in the plasma membrane composition negatively affect the function of many transport proteins localized in the membrane. In the next experiment, we measured the activity of the main MDR efflux pump in *C. glabrata*—*Cg*Cdr1p—using rhodamine 6G, an acknowledged substrate of efflux pumps energized by ATP (Izumikawa et al. 2003; Puri et al. 2011). As Fig. 4A shows, the energy-dependent rhodamine 6G efflux from the dye-preloaded *C. glabrata* cells was negligible in cells incubated in the presence of catechin. The increased susceptibility of *C. glabrata* cells to miconazole in the presence of catechin could thus be caused by the increased accumulation of miconazole inside cells as a result of its reduced efflux. The result obtained has been confirmed by the measurement of *CgCDR1* gene expression in the *C. glabrata lig4Δ* strain. Figure 4B demonstrates the mRNA expression of the *CgCDR1* gene in cells incubated in the presence of catechin or miconazole alone as well as in the combination of catechin with miconazole before the RNA extraction. The *CgCDR1* mRNA level in cells incubated in the presence of catechin was 5 times lower compared to that in control cells. On the other hand, miconazole induced the *CgCDR1* mRNA level more than 50 times (Fig. 4B). Incubation of cells in the common presence of both compounds—miconazole and catechin reverted the *CgCDR1* mRNA level to that observed in the control cells (Fig. 4B).

## Discussion

The natural tolerance of *Candida glabrata* to conventional antifungals and its strong capacity to acquire drug resistance make the infections caused by this pathogen particularly difficult to cure. The most widely used antifungals in the clinic are azoles, which inhibit fungal growth by disrupting ergosterol biosynthesis (Odds et al. 2003). The activity of azoles is fungistatic and leads to an increasing prevalence of resistance that is typically driven by pump-mediated drug efflux or by mutations in the drug target *ERG11* gene encoding lanosterol 14-α demethylase (Cowen et al. 2014). In the treatment of bacterial infections, combinational therapy has proven very effective and slowed the emergence of resistance (reviewed in Wu and Brown 2021). Several studies pointed also to a potential synergistic effect of the combination of antifungal azoles with plant-derived flavonoids (Hirasawa and Takada 2004; da Silva et al. 2014; Ning et al. 2015).

The present study shows the antifungal activity of the combination of catechin with miconazole against *C. glabrata* strain characterized at the laboratory level (*Cglig4Δ*) and clinical *C. glabrata* isolates resistant or sensitive to antifungal azoles. Catechin, while unable to inhibit the growth of *C. glabrata* itself, potentiates the antifungal activity of fluconazole and miconazole. We showed that the combined use of these antifungal azoles with catechin inhibited the growth of analysed *C. glabrata* strains. The combined use of fluconazole or miconazole with catechin was effective even against azole-resistant *C. glabrata* clinical isolate.

Flavonoids can exert both antioxidant and prooxidant activity (Yin et al. 2009; Suh et al. 2010; Hwang et al. 2012; Eghbaliferiz and Iranshahi 2016). Miconazole inhibits fungal peroxidase and catalase activities, while not affecting NADH oxidase activity, leading to increased production of ROS (Francois et al. 2006). The treatment of *C. glabrata* strains with miconazole or catechin alone in this study promoted the intracellular accumulation of ROS. The combined use of both compounds resulted in an additive effect. The intracellular ROS production was highest when the *C. glabrata* strains were treated with miconazole and catechin simultaneously.

Catechin containing aromatic rings in its structure can penetrate the phospholipid membranes due to the hydrophobic nature of the molecule (Daglia 2012). DPH and TMA-DPH are the most commonly used fluorescent probes to study the dynamical and structural properties of lipid bilayers and cellular membranes via measuring steady-state or time-resolved fluorescence anisotropy. Our steady-state fluorescence anisotropy measurements showed a significant decrease in fluorescence anisotropy of TMA-DPH and DPH embedded in the catechin-containing membrane, suggesting a substantial increase in membrane fluidity, which indirectly indicates a decrease in the order of the hydrocarbon chains. The altered membrane environment could be the basis for the observed reduced activity of the main drug efflux pump *Cg*Cdr1p. Thus, the inhibition of drug efflux ABC transporters may additionally contribute to the increased effectiveness of azole drugs. Our experimental evidence indicates that the incubation of cells in the presence of catechin leads to decreased expression of the *CgCDR1* gene. Although the presence of miconazole significantly induced the expression of the *CgCDR1* gene (more than fifty times compared to the control), *CgCDR1* gene expression was repressed in cells treated with the combination of miconazole and catechin. We propose that the generation and intracellular accumulation of ROS lead to defects in the structure and function of the plasma membrane and this affects the function of membrane-associated proteins. While the specific mechanism by which catechins exert synergy with antifungal drugs is not yet fully established, it is clear that the combination of the specific catechin used and antifungal drugs provides a promising approach to improve the treatment of resistant *C. glabrata* strains.
